# Implications of phase difference and amplitude ratio from intracochlear and extracochlear electrocochleography in normal-hearing guinea pigs

**DOI:** 10.3389/fnins.2026.1691034

**Published:** 2026-02-03

**Authors:** Imogen A. M. L. van Beurden, Dyan Ramekers, Robert J. Stokroos, Hans G. X. M. Thomeer, Huib Versnel

**Affiliations:** 1 Department of Otorhinolaryngology and Head & Neck Surgery, University Medical Center Utrecht, Utrecht University, Utrecht, Netherlands; 2 UMC Utrecht Brain Center, Utrecht University, Utrecht, Netherlands; 3 Department of Otorhinolaryngology Head and Neck Surgery, Antwerp University Hospital, Edegem, Belgium

**Keywords:** amplitude ratio, cochlear implantation, cochlear microphonic, cochlear trauma, electrocochleography, hearing preservation, phase difference

## Abstract

**Introduction:**

Electrocochleography (ECochG) can be performed extracochlearly from the round window, as well as intracochlearly from an electrode array as used in cochlear implantation. Cochlear microphonic (CM) amplitude ratio and phase difference from two fixed intracochlear electrodes may improve insight into local cochlear trauma after cochlear implantation.

**Methods:**

Six normal-hearing guinea pigs underwent cochleostomy and cochlear implantation. ECochG was recorded in response to pure tones between 0.25 and 16 kHz from one extracochlear site El_Ex and two intracochlear sites: El_Ap and El_Bs. The CM was analyzed in terms of threshold, amplitude ratio and phase difference. Regarding the analyses of the latter we focused on CM at 2 and 4 kHz. The compound action potential (CAP) was analyzed in terms of threshold and amplitude. Histological trauma and hair cell counts were determined in midmodiolar sections.

**Results:**

Both CM and CAP thresholds from El_Ap were approximately 5–10 dB lower than from El_Bs (CM thresholds: *p* = 0.003, CAP thresholds: *p* = 0.03). Across frequencies 0.25–8 kHz, CM amplitudes from El_Ap were a factor 4–8 larger than amplitudes from El_Ex (*p* < 0.001) and a factor 2–4 larger than amplitudes from El_Bs (*p* < 0.001). Across frequencies 0.25–8 kHz CAP amplitudes from El_Ap were a factor 2 larger than amplitudes from El_Bs (*p* = 0.02). The CM amplitude ratios between both intracochlear electrodes varied among animals from 1.7 to above 10 (median amplitude ratio: 2 kHz = 6.86; 4 kHz = 3.56), and CM phase differences varied from near 0 to −*π* (median phase difference: 2 kHz = −0.13π; 4 kHz = −0.32π). Four different combinations of small or large CM phase difference and small or large CM amplitude ratio were identified. In all animals, hair cell counts were normal. Histological trauma in the lateral wall around the site of cochleostomy was mild in four animals and moderate in two animals.

**Discussion:**

In addition to conventional electrophysiological thresholds, combinations of small or large CM phase difference and amplitude ratio from two intracochlear electrodes may mirror specific patterns of cochlear trauma as caused by cochlear implantation surgery.

## 1 Introduction

Cochlear implants (CIs) are highly effective in treating individuals with severe to profound sensorineural hearing loss. CIs result in significant improvement in speech perception, especially in quiet environments (Carlson, 2020; Gifford and Revit, 2010). Many patients eligible for cochlear implantation have some degree of residual hearing prior to implantation, particularly in lower frequencies (Bester et al., 2017; Kant et al., 2022; Lenarz et al., 2022). This residual acoustic hearing is often lost due to mechanical cochlear trauma inflicted by insertion of the CI’s electrode array into the scala tympani, an intracochlear inflammatory response or a change in basilar membrane (BM) mechanics (DeMason et al., 2012; Andonie et al., 2025; Bester et al., 2017; Sijgers et al., 2023).

Electrocochleography (ECochG) provides information about hair cell and cochlear nerve health during and after CI surgery, as it reflects electrophysiological responses of hair cells and the cochlear nerve to sound stimuli (Forgues et al., 2014; Harris et al., 2017). Summation of responses to stimuli of opposite phases reveals the neural component, the compound action potential (CAP), while reducing the hair cell component, the cochlear microphonic (CM). The CAP reflects auditory nerve fibers firing simultaneously in reaction to the onset and offset of a stimulus (Forgues et al., 2014; Eggermont, 2017; Fontenot et al., 2017). The CM reflects depolarization and hyperpolarization of the hair cell membrane potential, in response to BM movement. Subtraction of responses to stimuli of opposite phases reveals the CM, while reducing the CAP.

ECochG can be recorded extracochlearly from the round window, as well as intracochlearly from the electrode array of the CI (Eggermont, 2017). Previous animal and clinical studies have investigated intracochlear ECochG responses to detect cochlear trauma during CI surgery (Andrade et al., 2022; Bester et al., 2020; Campbell et al., 2017; Dalbert et al., 2021; Jwair et al., 2023; Sijgers et al., 2021; Suntinger et al., 2022). An animal study by Andrade et al. (2022) showed that electrode insertion had more severe impact on ECochG thresholds than cochleostomy. Jwair et al. (2023) induced various grades of trauma after CI in guinea pigs and found that responses to both high and low frequencies were affected with moderate cochlear trauma (including lateral wall damage at the cochleostomy) as well as with severe trauma (with additional damage to hair cells and the modiolar wall). With mild cochlear trauma, threshold shifts were limited to the region of the inserted electrode array (Andrade et al., 2022; Jwair et al., 2023).

Besides ECochG thresholds, intracochlear CM phase difference may also provide information about regional hair cell health near the recording electrode (Bester et al., 2020). Implications of CM phase difference for hair cell viability can be explained as follows: The travelling wave causes a place-dependent CM phase difference, which increases from base to apex in a healthy cochlea (Campbell et al., 2017; Robles and Ruggero, 2001). The absence of CM phase differences between CM recordings from two intracochlear recording sites suggests both recordings reflect the same population of hair cells, for instance because of hair cell damage at one of the recording sites (Campbell et al., 2017). Supporting this theory, Bester et al. found an increasing CM phase difference from base to apex in normal-hearing guinea pigs, whereas no CM phase differences were found in cochlear regions with elevated response thresholds, suggesting hair cell damage. Accordingly, in a clinical cohort, an absence of a CM phase difference between electrodes was associated with higher acoustic thresholds (Bester et al., 2020).

Intracochlear CM phase difference or amplitudes from multiple electrodes were further investigated in clinical studies by Dalbert et al. (2021), Sijgers et al. (2021), and Bester et al. (2023). Two studies compared CM data in terms of phase and amplitude changes from two electrodes; a fixed extracochlear one and an intracochlear one, which moved towards the apex during insertion of the electrode array. Both studies showed that analysis of CM amplitude and phase in intracochlear recordings from a single moving electrode is complex, as both acute cochlear damage and movement of the recording electrode along regions with different hair cell conditions contributed to different intracochlear response patterns (Dalbert et al., 2021; Sijgers et al., 2021). Bester et al. (2023) investigated the association of patterns of CM amplitudes from multiple fixed electrodes and preservation of residual hearing and found that subjects with a large intraoperative apical CM amplitude peak (where lower frequency residual hearing is located), had better postoperative residual hearing than the subjects with a basal amplitude peak. Therefore, we hypothesize that amplitudes and phase differences from stable responses from two fixed intracochlear electrodes, could provide additional insight into local hair cell viability (Dalbert et al., 2021; Sijgers et al., 2021; Suntinger et al., 2022; Bester et al., 2023).

In a previous study in guinea pigs, we examined the extent of the effect of acute surgical trauma during various stages of cochlear implantation on extracochlear recordings of CAP thresholds, amplitudes and latencies in relation to the degree of histological trauma (Jwair et al., 2023). Here, we investigate CAP data in terms of threshold and amplitude and CM data in terms of threshold, amplitude and phase obtained from one extracochlear electrode El_Ex and two intracochlear electrodes El_Ap and El_Bs in the same cohort as described in Jwair et al. (2023). We focus on inter-electrode phase differences and amplitude differences in relation to thresholds and histological trauma, to examine the additional value of the difference measures for assessment of local hair cell viability.

## 2 Materials and methods

### 2.1 Animals, surgical procedures and experimental design

The study population consisted of six female albino normal-hearing guinea pigs (20JWA28–20JWA33), obtained from Envigo (Horst, The Netherlands). Animals were kept under general laboratory conditions (food and water ad libitum; temperature of 21 °C; 60% humidity; lights on from 7:00 a.m. till 7:00 p.m.). Animals and surgical procedures for this experiment were identical to those detailed in Jwair et al. (2023), with all surgical and experimental procedures approved by the Animal Experiments Committee of Utrecht University (4315-1-01) and the Central Authority for Scientific Procedures on Animals (AVD1150020174315).

**Figure 1:**
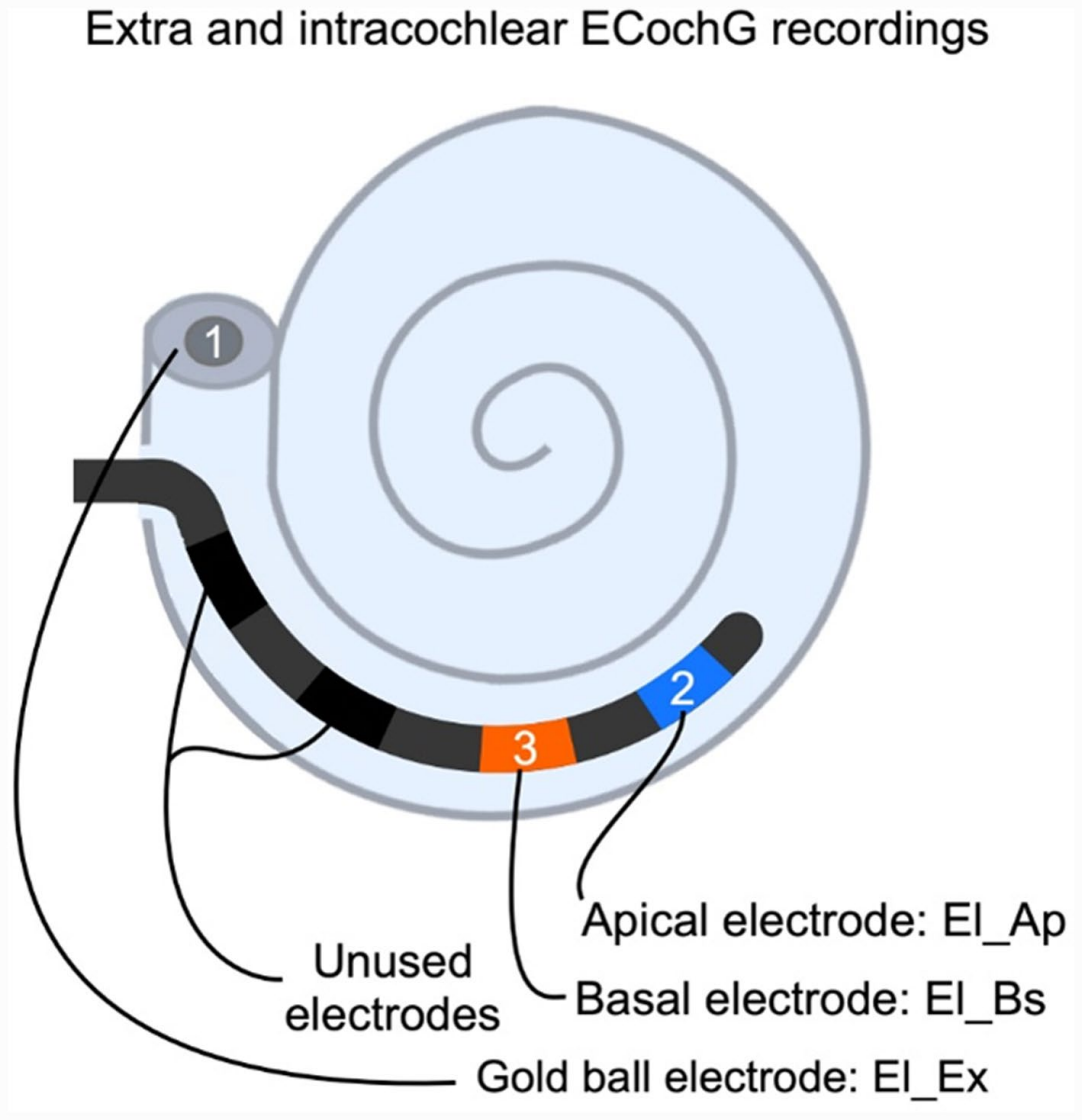
A schematic overview of the experimental setup, showing the three different electrodes El_Ex, El_Bs and El_Ap used to record electrocochleography (ECochG). After cochleostomy and insertion of the electrode array, the gold ball electrode El_Ex (1) is used to record extracochlear ECochG. Second, the apical electrode El_Ap (2) is used for intracochlear ECochG. Third, the basal electrode El_Bs (3) is used for intracochlear ECochG.

All animals were anesthetized using intramuscular injected dexmedetomidine (Dexdomitor; Vetoquinol, Breda, The Netherlands; 0.13 mg/kg) and ketamine (Narketan; Vetoquinol, Breda, The Netherlands; 20 mg/kg). The guinea pigs were tracheostomized to artificially ventilate the animals (1%–2% isoflurane in 1:2 O_2_ and N_2_O) during the entire experiment. Needle electrodes for auditory brainstem (ABR) recordings were positioned subcutaneously behind the right ear (active electrode) and subcutaneously in the center of the skull (reference electrode). For ECochG recordings, a transcranial screw, located 1 cm anterior from bregma, was used as recording reference electrode and a needle situated in the left hind limb of the animal functioned as ground electrode. The bony bulla was cleared from the neck muscles with an incision from the anterior medial side of the skull, towards retro-auricula from the right ear. To show the cochlear basal turn, the bulla was opened with a bullostomy. The gold ball electrode (stainless steel wire with 0.175 mm in diameter, a ball of 0.5 mm diameter fitted at the tip; Advent, Halesworth, UK) was positioned in the round window niche. A cochleostomy (CO) was made with a small hand drill with a diameter of 0.5 mm, and was located approximately 0.5 mm below the round window in the basal turn of the cochlea. The procedure was followed by insertion of a custom made electrode array (a diameter of 0.5 mm, tip-to-base array length of 3.5 mm; Advanced Bionics, Valencia, CA, USA), containing four electrodes with an interelectrode distance of 1.0 mm. Array insertion depth into the scala tympani amounted to approximately 4 mm. For an overview of the experimental set-up, see Figure 1.

### 2.2 Intra- and extracochlear electrocochleography recordings

ECochG was recorded from three different sites. Extracochlear recordings were performed with the gold-ball electrode, labelled El_Ex, positioned in the RW niche, after CO and cochlear implantation. Intracochlear recordings were performed using two adjacent electrodes of the electrode array. The electrode near the tip of the array was labelled El_Ap, the more basally located electrode was labelled El_Bs. El_Bs and El_Ap were approximately located at 3 and 4 mm from the round window, see Figure 1. Based on the cochlear frequency map of the guinea pig these locations correspond to characteristic frequencies of 22 and 16 kHz (Tsuji and Liberman, 1997).

As described in Jwair et al. (2023), measurements were conducted in a sound-attenuated room, with stimuli presented through a Bowers and Wilkens speaker (CCM683; 8 *Ω*; 25–130 W), positioned at 10 cm distance from the right pinna. Guinea pigs were exposed to seven pure tone stimuli at frequencies ranging from 0.25 kHz to 16 kHz, with one-octave steps/intervals. The tones were presented with alternating polarity, i.e., starting with either positive or negative phase. Time intervals between stimulus onsets were 99 ms. A minimum of two periods of rise-fall time and a minimum of two periods of plateau were chosen (Havenith et al., 2013). The duration of the tones decreased as stimuli increased in frequency. Rise/fall times also differed between frequencies. Stimuli of 250 Hz had a duration of 24 ms with a rise/fall time of 8 ms. Stimuli of 500 Hz tone had a duration of 12 ms, with a rise/fall time of 4 ms. The 1 kHz and 2 kHz stimuli had duration of 8 ms, and rise/fall time of, respectively, 2 ms and 1.5 ms. High frequency stimuli (4–16 kHz) had a duration of 8 ms with a rise/fall time of 1 ms. Maximum sound levels (in dB SPL), differed between each frequency and were 99 dB SPL at 0.25 and 0.5 kHz, 103 dB SPL at 1 kHz, 98 dB SPL at 2 kHz, 104 dB SPL at 4 kHz, 110 dB SPL at 8 kHz and 107 dB SPL at 16 kHz. These maximum sound levels were subsequently attenuated gradually in decrements of 10 dB, until no CAP and CM responses could be identified. The signal was pre-amplified with a Princeton Applied Research (Oak Ridge, TN, United States) 5,113 pre-amplifier (amplification × 5,000; band pass filter 0.1–30 kHz). A TDT3 system was utilized for recording (100 kHz sampling rate, 24-bit sigma-delta converter). ECochG responses were averaged over a maximum of 500 repetitions. Measurements were stored on a PC for off-line analysis.

### 2.3 Histological processing

Once all electrophysiological measurements had been completed and the electrode array was removed, the guinea pigs were terminated using an overdose of pentobarbital injected intracardially. The cochleas were harvested and processed for histological analysis. First cochlear fixation was done using a fixative of 3% glutaraldehyde, 2% formaldehyde, 1% acrolein, and 2.5% dimethyl sulfoxide (DMSO) in a 0.08 M sodium cacodylate buffer. Fixation was followed by decalcification in 10% EDTA for approximately 10 days. The cochleas were fixed in 1% osmium tetroxide and 1% potassium ruthenium cyanide and finally embedded in Spurr’s low-viscosity resin. For histological staining 1% methylene blue, 1% azur B, and 1% borax in distilled water were used. Before analysis could be done, cochlear tissues were sectioned into five midmodiolar sections of 1 μm each, using a Leica RM2265 microtome. The sections were put on a slide with coverslip and were kept in a sequential order (Jwair et al., 2023).

### 2.4 Data analysis

**Figure 2:**
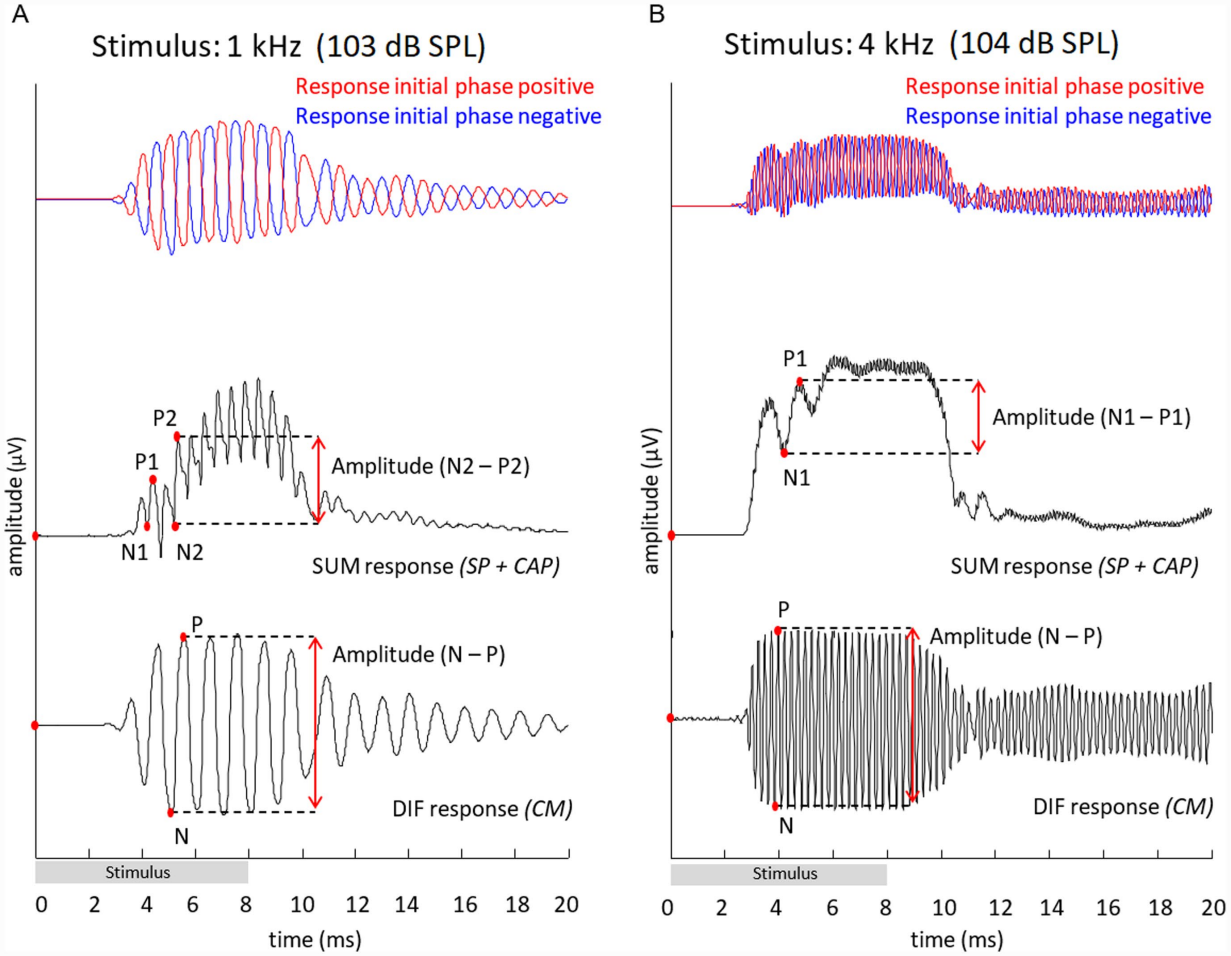
Examples of electrocochleography to two tones, 1 kHz **(A)** and 4 kHz **(B)**, measured in animal 20JWA28. Responses are shown at maximum sound levels (0 dB attenuation). Stimuli had a duration of 8 ms. The SUM response, also called compound action potential (CAP), was generated by adding up the two responses to opposite phase (shown in red and blue). At low frequencies (0.25 kHz – 2 kHz), peak-to-peak CAP amplitudes were calculated between points N2 and P2. For high frequency tones (4 kHz – 16 kHz), peak-to-peak CAP amplitudes were calculated between N1 and P1. The DIF response, mainly consisting of the cochlear microphonics (CM), was identified by subtracting the responses to the opposite phase.

Analysis of ECochG responses was done using custom-written MATLAB scripts (MATLAB version R2024b). Figure 2 provides two examples of pairs of ECochG waveforms, in response to a low frequency tone (1 kHz) and a high frequency tone (4 kHz) both at maximum sound levels (103 and 104 dB SPL, respectively), measured in one individual animal 20JWA28. As tones were presented with alternating polarity, responses with an initial positive and an initial phase negative were generated. By summing these responses, creating the SUM response, the CAP was analyzed. By subtracting both initial phase positive and initial phase negative responses, the DIF response was generated, which mainly consists of the CM.

**Figure 3:**
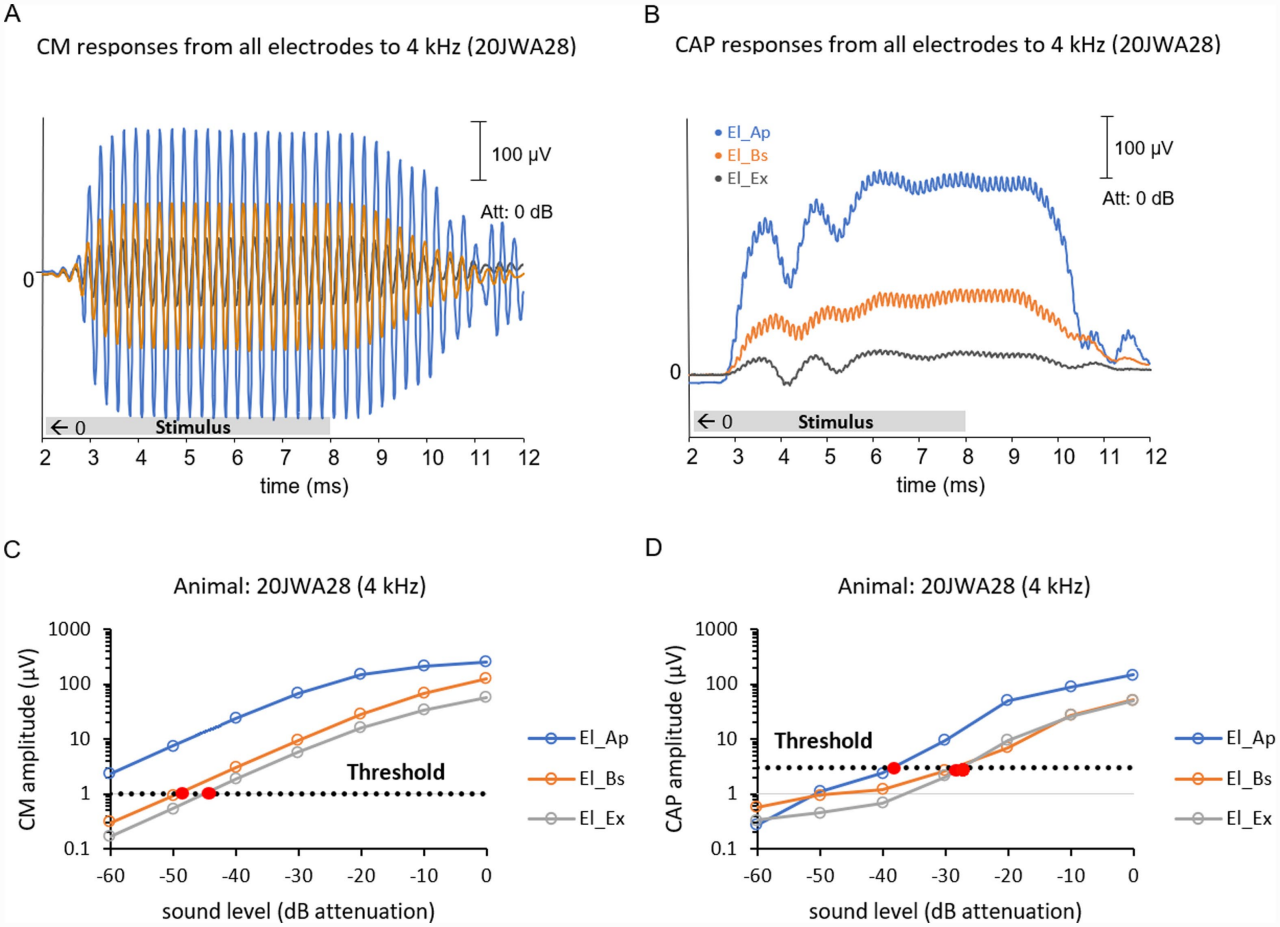
Example of CM responses **(A)** and CAP responses **(B)** measured from all three electrodes: extracochlear electrode El_Ex (in dark grey), apical intracochlear electrode El_Ap (in blue) and basal intracochlear electrode El_Bs (in orange). Responses are from one individual animal 20JWA28 and to a pure tone of 4 kHz at maximum sound level (104 dB SPL). CM and CAP thresholds were calculated through interpolation at pre-set threshold criteria. Above examples of input/output curves from one individual animal 20JWA28 show CM and CAP amplitude as function of sound level in dB attenuation in response to a pure tone of 4 kHz. Dashed lines show the threshold criteria of 3 μV for CAP thresholds **(C)** and the threshold criteria of 1 μV for CM thresholds **(D)**.

Corresponding cochlear microphonics in response to all frequencies 0.25–16 kHz were generated using the same custom written MATLAB scripts. Using fast Fourier transforms (FFT) of the response in a window where the response plateaus the amplitude and phase were derived. Figure 3A exhibits an example of CM responses El_Ex, El_Ap and El_Bs at a pure 4 kHz tone. CM thresholds were calculated through interpolation at a pre-set threshold criterion of 1 μV, as shown in Figures 3C,D. In case the response at the lowest level recorded was above the criterion, threshold was estimated through extrapolation. Input/output curves were generated showing CM amplitude as a function of sound level, as shown in an example in Figure 3A. CM amplitude ratios at approximately 90 dB SPL were calculated as follows: amplitudes El_Ap/El_Bs, amplitudes El_Ap/El_Ex and amplitudes El_Bs/El_Ex. CM phase differences were calculated at the same level by subtracting phases of El_Bs from El_Bs, El_Ex from El_Bs and El_Ex from El_Ap.

Figure 3B exhibits an example of CAP responses from all electrodes El_Ex, El_Ap and El_Bs at a pure 4 kHz tone. Input/output curves were generated showing CAP amplitude as a function of sound level, as shown in an example in Figure 3D. CAP thresholds were calculated through interpolation at a pre-set threshold criterion of 3 μV for high frequencies (4–16 kHz) and 1 μV for low frequencies (0.25–2 kHz), as shown in Figure 3D.

For CAP responses to low frequency tones (0.25–2 kHz), the first negative dip (N) in the response was chosen to be N1, with the following peak (P) to be P1. N1-P1 was calculated as the peak-to-peak amplitude. A second peak-to-peak amplitude was determined by selecting a lowest point (N2) and a subsequent highest point (P2) in the curve, which typically was the largest amplitude in the series of N-P peaks. At high frequency tones (4–16 kHz), only one peak-to-peak amplitude was determined. In these select scenarios, the first lowest point (N1) and its following highest point (P1) were selected.

CAP amplitude ratios between both intracochlear electrodes El_Ap and El_Bs and extracochlear electrode El_Ex were calculated by dividing El_Ap by El_Bs, El_Ap by El_Ex and El_Bs by El_Ex. In order to obtain ratios at similar sound levels (approximately 90 dB SPL) for all frequencies, sound levels of −10 dB attenuation were chosen for frequencies 0.25 kHz – 4 kHz, and values measured at −20 dB attenuation were chosen for frequencies 8 kHz and 16 kHz.

In order to assess cochlear trauma, CM amplitude ratios of El_Ap/El_Bs were compared to CM phase differences between El_Ap and El_Bs. CM thresholds from El_Ap and El_Bs and CAP thresholds from El_Ex were also included in assessments of local hair cell health and cochlear trauma. Only responses to 2 and 4 kHz were utilized for these analyses for the following reasons. Frequencies 0.25–1 kHz were excluded as phase differences were small and the DIF response was often contaminated with neural components. Frequencies 8 and 16 kHz were also excluded from these analyses, as phase differences were large and suffering from 2π ambiguities. Thus, responses to 2 and 4 kHz were considered the most reliable to assess local hair cell health.

The phase difference in a healthy condition can be predicted based on group difference data in guinea pigs using *τ* = 88f^−0.486^ with τ the difference in ms and f frequency in Hz (Adel et al., 2021). Assuming the locations of the electrodes correspond to characteristic frequencies of 22 and 16 kHz the difference would be 0.11 ms. It is reasonable therefore to expect a difference above our criterion of 0.0625 ms in a healthy cochlea. Note that in theory the phase differences are negative. For CM phase difference a criterion of −0.25π was employed for 2 kHz and −0.5π for 4 kHz (corresponding to a difference of 0.0625 ms). Phase difference above the criterion were considered small and below the criterion were considered large. Phase differences that are considerably smaller than the criterion indicate trauma (Campbell et al., 2017) but also phase differences that are considerably larger may indicate trauma. For CM amplitude ratio, a criterion of 3.5 was employed, based on visual inspection of CM responses to 2 and 4 kHz at −10 dB attenuation (88 and 94 dB SPL, respectively), in each case showing larger responses for Ap than for Bs. CM amplitude ratios <3.5 were considered small and ratios ≥3.5 were considered large.

**Figure 4:**
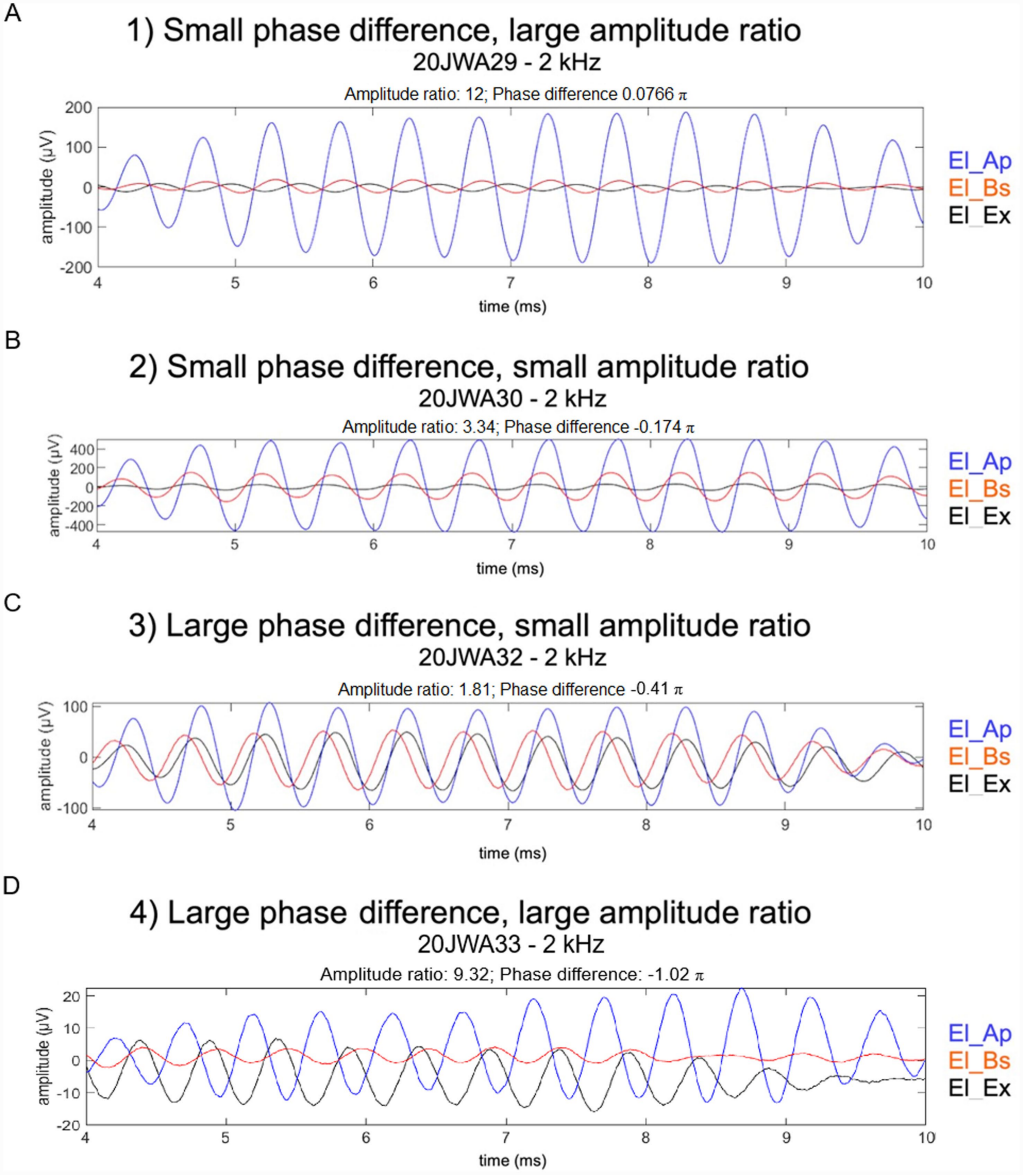
CM responses to 2 and 4 kHz at 88 and 94 dB SPL, respectively, from all animals were categorized into one of the following four categories. **(A)** 1) A small CM phase difference (−0.25–0.25 *π* for 2 kHz and −0.5 – 0.5 π for 4 kHz) with a large amplitude ratio (ratio ≥ 3.5). **(B)** 2) A small CM phase difference and amplitude ratio (ratio ≤ 3.5). **(C)** 3) A large phase difference (<−0.25 π or >0.25 π for 2 kHz and <−0.5 π or >0.5 π for 4 kHz) in combination with a small amplitude ratio (ratio ≤ 3.5). **(D)** 4) A large CM phase difference and a large amplitude ratio (ratio ≥ 3.5).

After assessment based on these criteria, responses to 2 and 4 kHz for all animals could be categorized into one of the following for categories: (1) a small CM phase difference (−0.25–0.25 for 2 kHz and −0.5 – 0.5 for 4 kHz) with a large amplitude ratio (ratio ≥ 3.5); (2) a small CM phase and amplitude ratio (ratio ≤ 3.5); (3) a large phase difference (<−0.25 or >0.25 for 2 kHz and <−0.5 or >0.5 for 4 kHz) in combination with a small amplitude ratio (ratio < 3.5); and (4) a large CM phase difference and a large amplitude difference (ratio ≥ 3.5). Figure 4 shows examples of each category, based on CM responses to 2 or 4 kHz.

### 2.5 Histological trauma assessment

One midmodiolar section per cochlea was macroscopically assessed to determine the amount of trauma. Trauma severity was rated based on: fracture of modiolar wall (yes or no), OSL fracture (yes or no), and lateral wall damage around cochleostomy (as expected: +, more traumatic: ++) (Jwair et al., 2023). Evaluation of hair cell structural integrity (e.g., dislocated or abnormally shaped hair cells) was assessed, and quantification of inner and outer hair cells from cochlear base to apex was performed. In the case of abnormal structural integrity of hair cells, animals were rated positive for hair cell damage. Histological trauma assessment for animals 20JWA28-30 and JWA32-33 was obtained from analysis by Jwair et al. (2023). Sections from animal 20JWA31 were newly assessed for this study, as Jwair et al. (2023) excluded this animal as it had high CAP thresholds before cochleostomy and was considered to have hearing loss before the start of the experiment; for the current study that suspected hearing loss was not relevant and therefore it could be included.

### 2.6 Statistics

Repeated-measures ANOVA (RM ANOVA) in IBM SPSS Statistics (SPSS version 30.0.0.0) was used to examine main effects of frequency and electrode, and interaction effects of these factors, on CAP and CM thresholds and amplitudes. Results were expressed as F-statistics (F) with degrees of freedom (df) and *p*-values (P). Within the same RM ANOVA, simple contrasts was used for comparisons between two electrodes. In all cases a *p*-value < 0.05 was considered statistically significant.

## 3 Results

### 3.1 Cochlear microphonics

#### 3.1.1 Threshold

**Figure 5:**
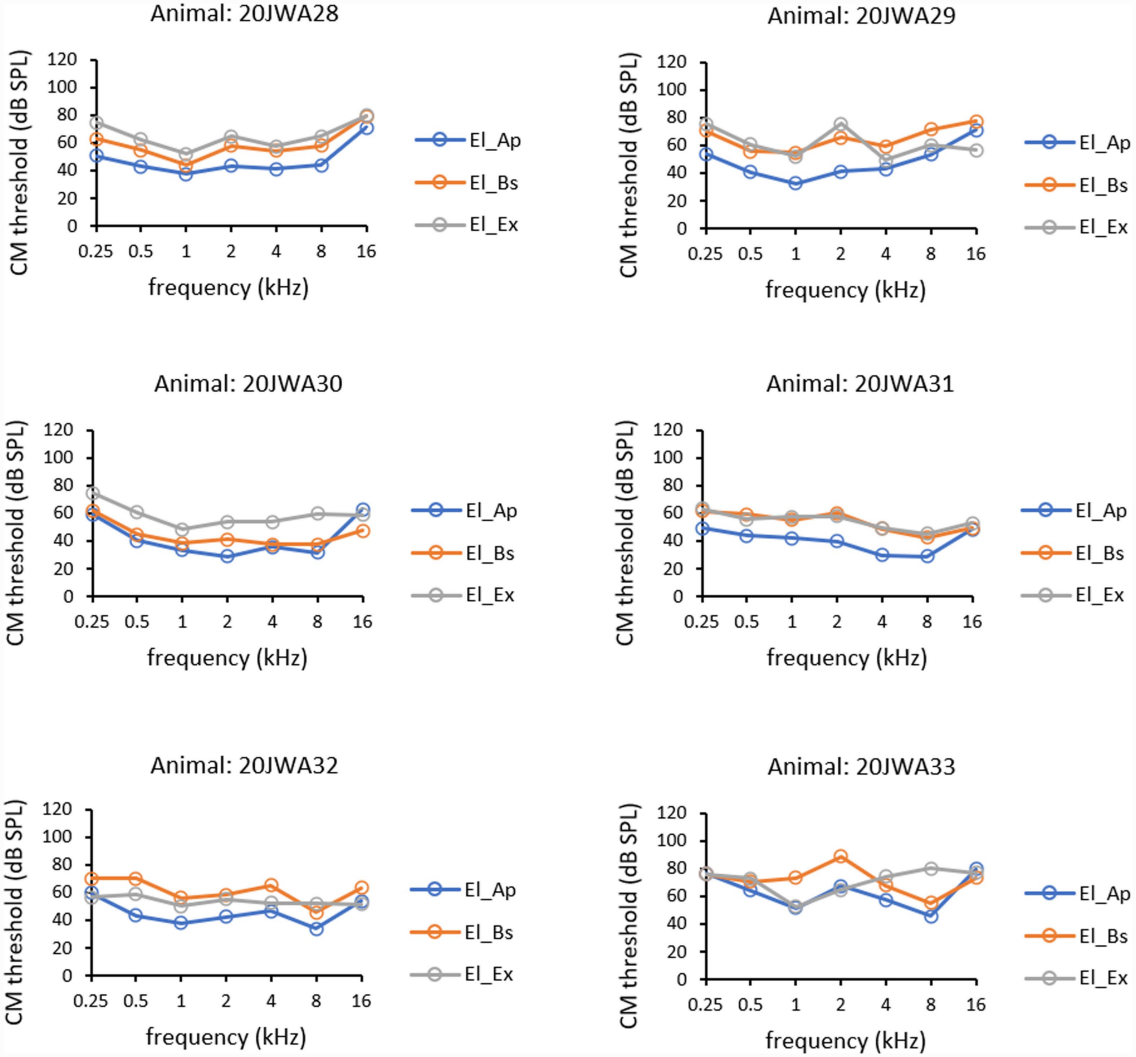
CM thresholds for all three electrodes: extracochlear electrode El_Ex (dark grey), apical intracochlear electrode El_Ap (blue), and basal intracochlear electrode El_Bs (orange). Thresholds at all frequencies 0.25–16 kHz are shown from all six animals.

CM thresholds (in dB SPL) as a function of tone frequency (0.25–16 kHz) of all individual animals for the three electrode sites are depicted in Figure 5. Outcomes of repeated measures (RM) ANOVAs for CM thresholds and amplitudes are listed in Table 1. In all animals the lowest thresholds were measured intracochlearly. The effect of electrode location on CM thresholds was statistically significant (*F*_(2, 10)_ = 17.45; *p* < 0.001). A known main effect was found of frequency with the lower thresholds for the middle frequencies (*F*_(2.29, 11.4)_ = 9.31; *p* = 0.003). No significant interaction effect on CM thresholds was seen between frequency and electrode location (*F*_(1.93, 9.66)_ = 3.03; *p* = 0.10). Thresholds from El_Ap were approximately 5–10 dB lower than from El_Bs (RM ANOVA, p = 0.003). Thresholds measured from El_Ap were approximately 10–20 dB lower than from El_Ex (*p* = 0.001). No significant difference was observed between basal electrode El_Bs and extracochlear electrode El_Ex (*p* = 0.60).

**Table 1:** CM: results from repeated measures ANOVAs.

CM		Main effects		Interaction effects	Between pair analysis		
		0.25–16 kHz		0.25–16 kHz	0.25–16 kHz		
		Electrode^a^	Frequency^b^	Frequency × electrode^b^	El_Ap vs. El_Bs	El_Ap vs. El_Ex	El_Bs vs. El_Ex
Threshold	F	17.45 (2)	9.31 (2.29)	3.03 (1.93)	—	—	—
	P	<0.001*	0.003*	0.10	0.003*	<0.001*	0.60
Amplitude	F	29.87 (2)	18.20 (2.16)	4.20 (2.58)	—	—	—
	P	<0.001*	<0.001*	0.03*	<0.001*	<0.001*	0.21

CM, cochlear microphonics; El_Ap, apical intracochlear electrode; El_Bs, basal intracochlear electrode; El_Ex, extracochlear electrode.

a Sphericity assumed.

b Greenhouse-Geisser corrected.

Degrees of freedom (df) given in brackets, *p* = *p*-value, with 0.05 significance level*.

#### 3.1.2 Amplitude

**Figure 6:**
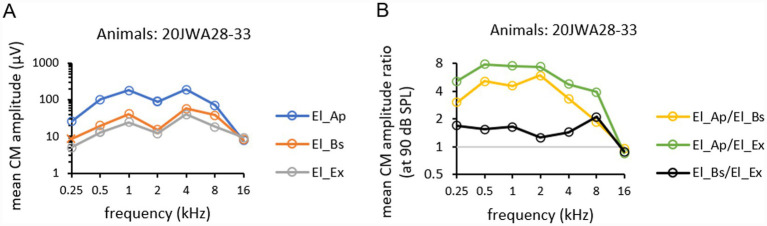
Mean CM amplitudes at ~90 dB SPL across all frequencies 0.25–16 kHz **(A)** were calculated from CM amplitudes from six individual animals 20JWA28–33. Mean CM amplitude ratios at ~90 dB SPL across all frequencies **(B)** were calculated by dividing amplitudes from El_Ap/El_Bs (yellow), El_Ap/El_Ex (green), and El_Bs/El_Ex (black).

Mean CM amplitudes of all six animals, as function of tone frequency (0.25–16 kHz) obtained at ~90 dB SPL and recorded from the three electrodes are depicted in Figure 6A. Mean CM amplitude ratios for all three electrodes, El_Ap, El_Bs and El_Ex, as a function of all tone frequencies (0.25–16 kHz) obtained at ~90 dB SPL are depicted in Figure 6B. The effect of electrode location on CM amplitudes was significant (*F*_(2,10)_ = 29.87; *p* < 0.001). Also, frequency had a significant effect on CM amplitude (*F*_(2.16, 10.8)_ = 18.20, *p* < 0001) with values peaking at 1 and 4 kHz. At 16 kHz no difference in amplitudes between electrodes was seen, thus no significant interaction effect was found between frequency and electrode (*F*_(2.58, 12.9)_ = 4.20, *p* = 0.03). Across frequencies 0.25–8 kHz amplitudes from El_Ap were a factor 2–4 larger than amplitudes from El_Bs (RM ANOVA, *p* < 0.001). Across frequencies 0.25–8 kHz amplitudes from El_Ap were a factor 4–8 larger than amplitudes from El_Ex (*p* < 0.001). No significant difference was found between amplitudes from the basal electrode El_Bs and those from the extracochlear electrode El_Ex (*p* = 0.21).

### 3.2 Compound action potential

#### 3.2.1 Threshold

**Figure 7:**
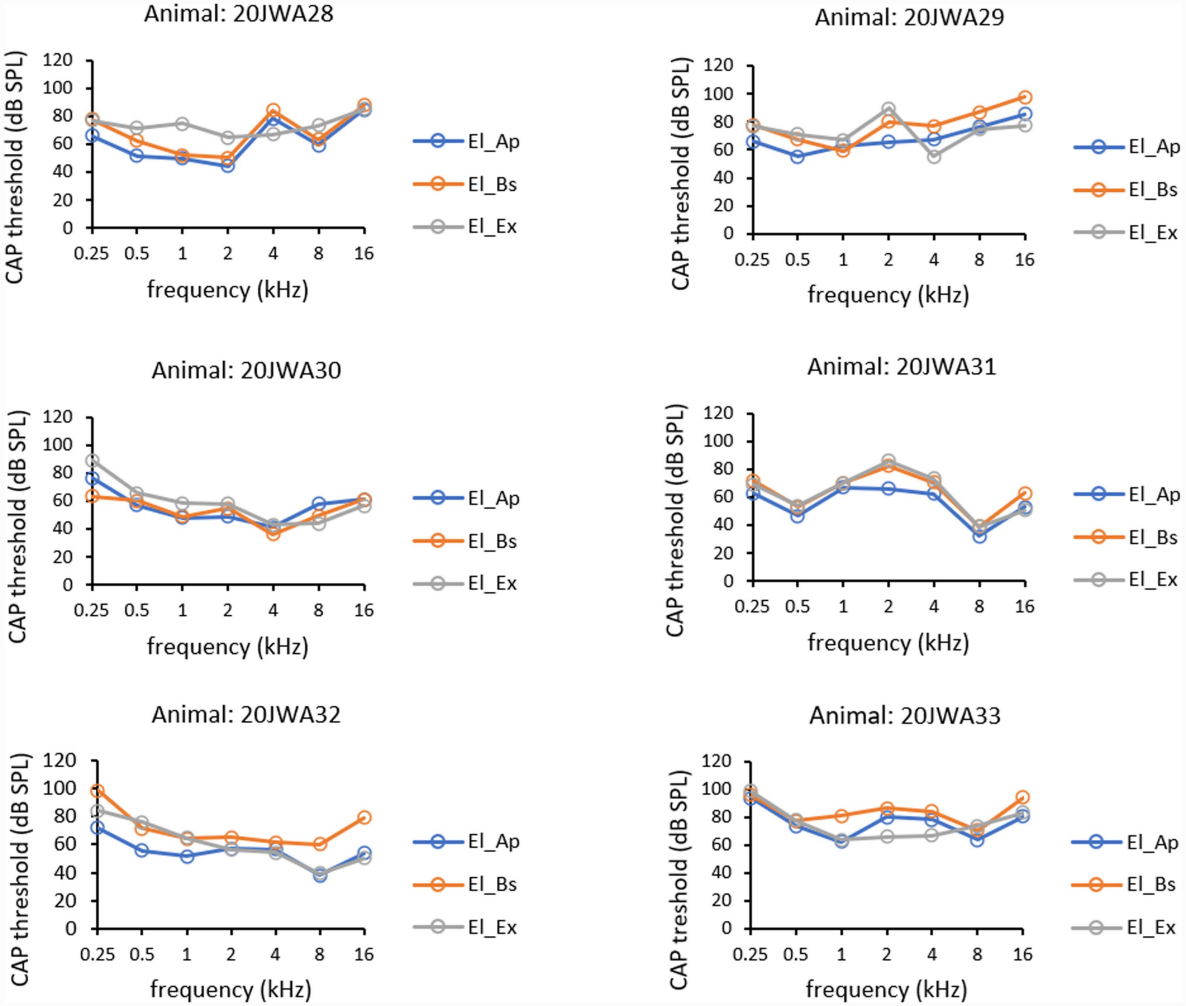
CAP thresholds for all three electrodes: extracochlear electrode El_Ex (dark grey), apical intracochlear electrode El_Ap (blue) and basal intracochlear electrode El_Bs (orange). Thresholds at all frequencies 0.25–16 kHz are shown from all six animals 20JWA28–33.

CAP thresholds (in dB SPL) as a function of tone frequency (0.25–16 kHz) of all individual animals are depicted in Figure 7. Outcomes of RM ANOVAs for CAP amplitudes are listed in Table 2. A pattern similar to that of CM thresholds was observed in CAP thresholds. Thresholds peaked at 0.25 and 16 kHz in animals 20JWA28-30, 32 and 33. In animal 20JWA31 (with suspected hearing loss at baseline) CAP thresholds peaked at 2 kHz. A significant effect of electrode on thresholds, was found with thresholds lowest measured from intracochlear El_Ap (*F*_(2,10)_ = 5.66, *p* = 0.02). The effect of frequency on CAP thresholds was not significant (*F*_(2.63,13.1)_ = 3.14, *p* = 0.07). No significant interaction effect was found between frequency and electrode (*F*_(3.60,18.0)_ = 2.72, p = 0.07). Thresholds from El_Ap were approximately 5–10 dB lower than from El_Bs (RM ANOVA, p = 0.03). Thresholds from El_Ap were approximately 10–15 dB lower than thresholds from El_Ex in frequencies 0.25–2 kHz (*p* = 0.02). No differences were seen between thresholds for El_Bs and El_Ex (*p* = 0.45).

**Table 2:** Results from repeated measures (RM) ANOVAs.

CAP		Main effects		Interaction effects	Between pair analysis		
		0.25–16 kHz		0.25–16 kHz	0.25–16 kHz		
		Electrode^a^	Frequency^b^	Frequency × Electrode^b^	El_Ap vs. El_Bs	El_Ap vs. El_Ex	El_Bs vs. El_Ex
Threshold	F	5.66 (2)	3.14 (2.63)	2.72 (3.60)	—	—	—
	P	0.02*	0.07	0.07	0.03*	0.02*	0.45
Amplitude	F	10.13 (2)	12.46 (1.94)	1.79 (3.42)	—	—	—
	P	0.004*	0.002*	0.18	0.02*	0.002*	0.23

CAP, compound action potential; El_Ap, apical intracochlear electrode; El_Bs, basal intracochlear electrode; El_Ex, extracochlear electrode.

a Sphericity assumed.

b Greenhouse-Geisser corrected.

Degrees of freedom (df) given in brackets, *p*, *p*-value, with 0.05 significance level*.

#### 3.2.2 Amplitude

**Figure 8:**
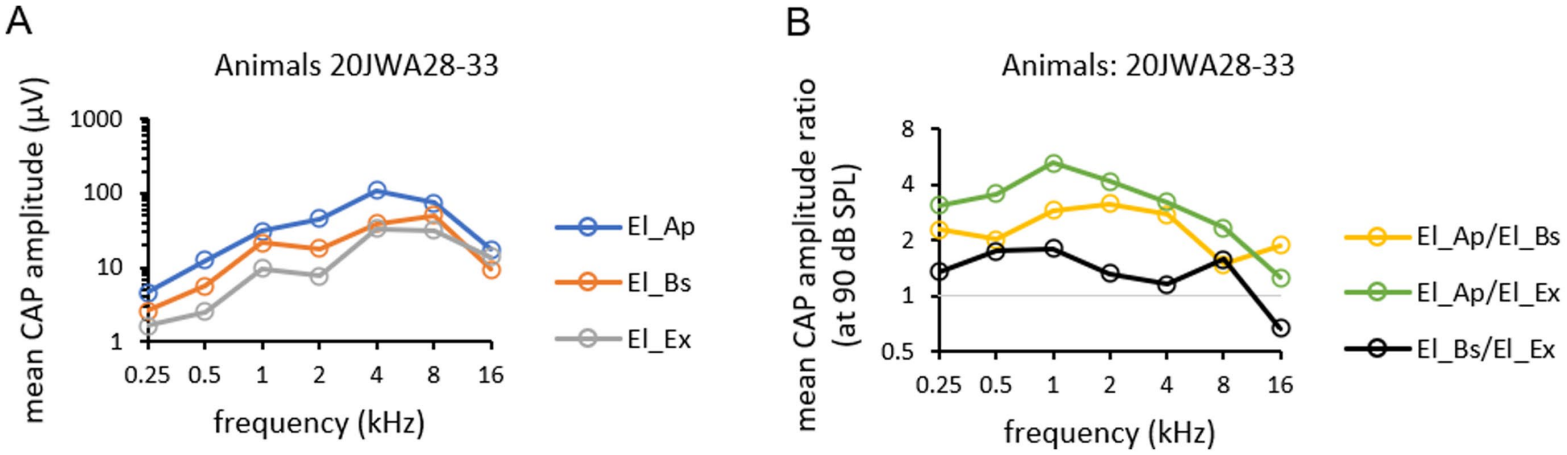
Mean CAP amplitudes at ~90 dB SPL across all frequencies 0.25–16 kHz **(A)** were calculated from CAP amplitudes from amplitudes from six individual animals 20JWA28-33. Mean CAP amplitude ratios at 90 dB SPL across all frequencies **(B)** were calculated by dividing amplitudes from El_Ap/El_Bs (yellow), El_Ap/El_Ex (green), and El_Bs/El_Ex (black).

Mean CAP amplitudes of all six animals, as function of tone frequency (0.25–16 kHz) obtained at ~90 dB SPL at the three electrodes are depicted in Figure 8A. Mean CAP amplitude ratios for all three electrodes, as a function of all tone frequencies (0.25–16 kHz) obtained at ~90 dB SPL are depicted in Figure 8B. Outcomes of repeated measures ANOVAs for CM amplitudes are listed in Table 2. Electrode localization had a significant effect on CAP amplitudes, with mean CAP amplitudes larger measured intracochlearly than measured extracochlearly (F_(2,10)_ = 10.13, *p* = 0.004). Frequency had a significant effect on CAP amplitudes with peak values at 4 and 8 kHz (*F*_(1.94, 9.70)_ = 12.46, *p* = 0002). No significant interaction effect on CAP amplitude was seen between frequency and electrode (*F*_(3.42, 17.1)_ = 1.79, *p* = 0.18). Across frequencies 0.25–16 kHz amplitudes from El_Ap were a factor 2 larger than amplitudes from El_Bs (RM ANOVA, *p* = 0.02). Across frequencies 0.25–4 kHz amplitudes from El_Ap were a factor 4 larger than amplitudes from El_Ex (*p* = 0.002). At 8 and 16 kHz the amplitudes from El_Ap, El_Bs and El_Ex differed a factor 1–1.5. No differences in amplitude were seen between El_Bs and El_Ex (*p* = 0.23).

### 3.3 Histological trauma assessment

Results of histological trauma assessment are listed in Table 3. All animals had a normal HC count, apart from 20JWA28, which had some OHC loss in middle segment and IHC loss in apical segment. None of the animals had osseous spiral lamina (OSL) or modiolar wall fractures. Thus, four animals (20JWA28-31) had a trauma severity rating of minimal, solely based on minimal lateral wall (LW) damage at the cochleostomy site. Two animals (20JWA32 and 33) had a trauma severity rating of moderate, as moderate LW damage at the cochleostomy site was observed.

**Table 3:** Results from histological trauma assessment.

Animal	Hair cell counts						
	IHC basal	OHC basal	IHC mid	OHC mid	IHC apex	OHC apex	Trauma severity rating
20JWA28	2/2	6/6	2/2	5/6	1/2	6/6	Minimal*
20JWA29	2/2	6/6	2/2	6/6	2/2	6/6	Minimal*
20JWA30	2/2	6/6	2/2	6/6	2/2	6/6	Minimal*
20JWA31	2/2	6/6	2/2	6/6	2/2	6/6	Minimal*
20JWA32	2/2	6/6	2/2	6/6	2/2	6/6	Moderate**
20JWA33	2/2	6/6	2/2	6/6	2/2	6/6	Moderate**

IHC, inner hair cell; OHC, outer hair cell.

Normal-hearing guinea pig cochleas count 2 IHCs and six OHCs per section (basal, mid, apex).

*Based on normal lateral wall damage at cochleostomy (+). No hair cell damage, OSL or modiolar wall fracture.

**Based on moderate lateral wall damage at cochleostomy (++). No hair cell damage, OSL or modiolar wall fracture.

### 3.4 CM phase differences and amplitude ratios in relation to thresholds and histological trauma

**Figure 9:**
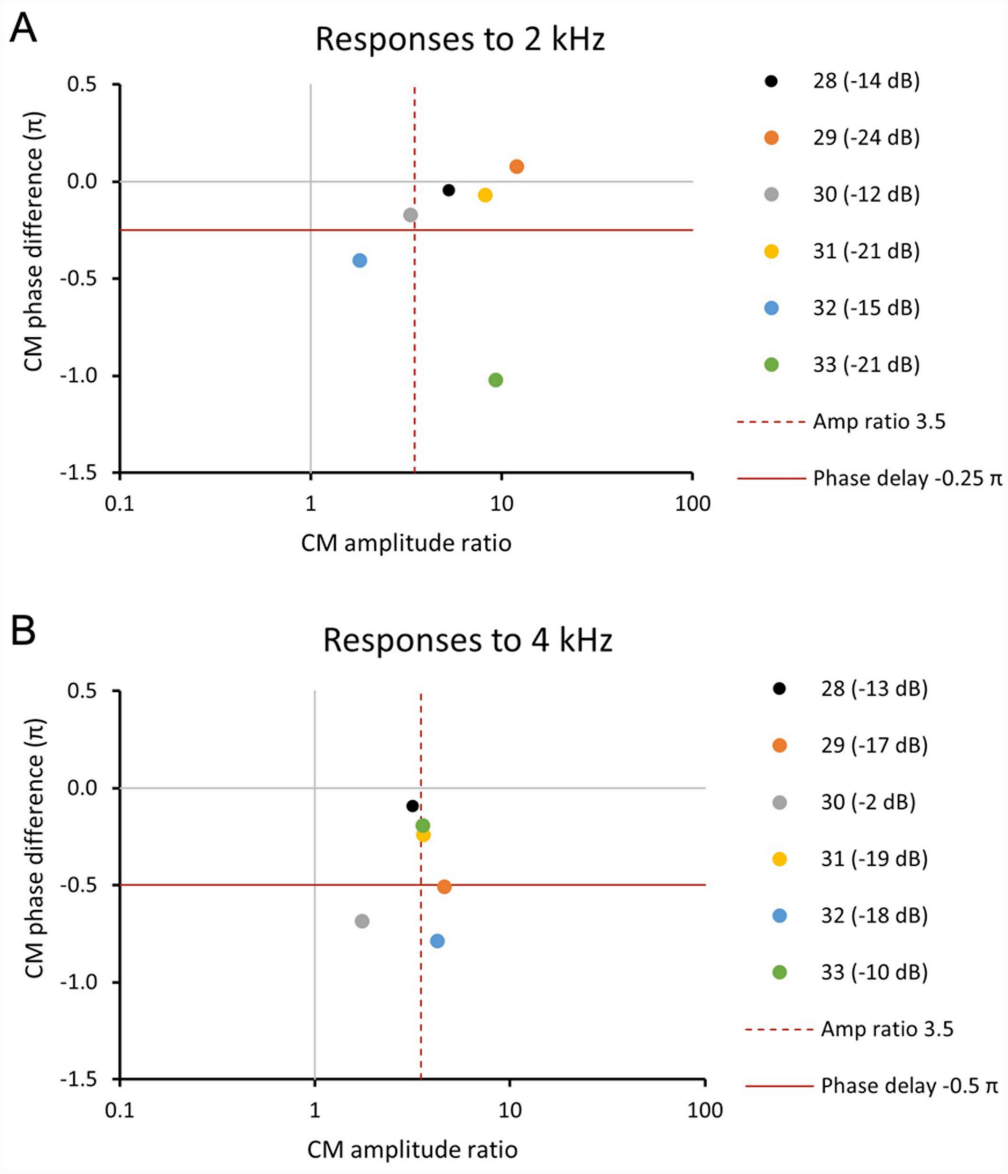
Scatter plots of CM phase difference El_Ap – El_Bs versus CM amplitude ratio El_Ap/El_Bs for all six animals at frequencies 2 kHz **(A)** and 4 kHz **(B)**, with differences in CM threshold between El_Ap and El_Bs added beside each animal in the legend.

Comparison of CM phase difference El_Ap – El_Bs and CM amplitude ratio El_Ap/El_Bs for all six animals at frequencies 2 and 4 kHz are listed in Table 4. The results of this comparison are visualized in scatterplots in Figure 9, with differences in CM thresholds between El_Ap and El_Bs added as labels of each point in the plot. Based on the criteria for CM phase difference and CM amplitude ratio (see Methods for criteria), four different combinations were found: (1) −/+ a small CM phase difference with a large amplitude ratio (2) −/−; a small CM phase and small amplitude ratio, (3) +/−; a large phase difference in combination with a small amplitude ratio and (4) +/+; a large CM phase difference and a large amplitude ratio (ratio ≥ 3.5).

**Table 4:** Results from comparison of CM phase difference and CM amplitude ratio.

Animal	Frequency (kHz)									
	2					4				
	∆ *φ*	Amp ratio	Thresholds (dB)			∆ φ	Amp ratio	Thresholds (dB)		
			CM Ap	CM Bs	CAP Ex			CM Ap	CM Bs	CAP Ex
20JWA28	−	+	44	58	65	−	−	41	54	67
20JWA29	−	+	41	66	90	+	+	43	59	55
20JWA30	−	−	29	41	58	+	−	36	38	43
20JWA31	−	+	40	60	86	−	+	30	49	73
20JWA32	+	−	43	58	56	+	+	47	65	54
20JWA33	+	+	67	89	66	−	+	57	68	67

∆ φ, CM phase difference; Amp ratio, CM amplitude ratio El_Ap/El_Bs; CM Ap, CM threshold El_Ap; CM Bs, CM thresholds El_Bs; CAP Ex, CAP threshold El_Ex.

–, amplitude ratio ≤3.5; −, phase difference −0.25 – 0.25 for 2 kHz; −, phase difference −0.5 – 0.5 for 4 kHz; +, amplitude ratio ≥3.5; +, phase difference <−0.25 or >0.25 for 2 kHz; +, phase difference <0.5 or >0.5 for 4 kHz.

Colours: (1) light green = small phase difference, large amplitude ratio, (2) dark green = small phase and amplitude ratio, (3) light grey = large phase difference, small amplitude ratio, (4) dark grey = large phase and amplitude ratio.

At 2 kHz (Figure 9A), all six animals showed amplitude ratios larger than 1, with values ranging between 1.8 and 12 (median amplitude ratio = 6.86; median phase difference = −0.13π). For 5 animals (20JWA28-32) the phase difference decreased with increasing amplitude ratio. In turn, the amplitude ratio tended to increase with threshold difference, see Figure 9A and Table 4. In three animals (20WA28, 29, 31) a small CM phase difference (above −0.25π) and a large amplitude ratio (≥3.5) was found. These animals were rated in the minimal trauma group (Table 3). Only two animals 20JWA32 and 33 showed large phase differences: Animal 20JWA32 showed a large phase difference and a small amplitude ratio, with a difference in CM thresholds of −15 dB. Animal 20JWA33 showed a large phase difference and large amplitude ratio, together with a difference in CM thresholds of −21 dB. 20JWA32 and 33 were the only animals with a moderate trauma severity rating (based on lateral wall damage at the cochleostomy site), but overall thresholds in 20JWA32 were relatively low (CM Ap = 43 dB SPL; CM Bs = 58 dB SPL; CAP Ex = 56 dB SPL). Overall thresholds in 20JWA33, with moderate histological trauma, were the highest of all animals (CM Ap = 67 dB SPL; CM Bs = 89 dB SPL; CAP Ex = 66 dB SPL).

At 4 kHz, all six animals also showed amplitude ratios larger than 1, with values ranging between 1.7 and 4.6 (median amplitude ratio = 3.56; median phase difference = −0.32π). Similar amplitude ratios were seen in five animals 20JWA28, 33, 31, 29 and 32. The largest differences between CM thresholds from El_Ap and El_Bs (ranging between −10 dB and −19 dB) were seen in animals 20JWA33, 31, 29 and 32 with amplitude ratios larger than 3.5. Histological trauma in animal 20JWA33 was rated moderate and overall thresholds were the highest measured (CM Ap = 57 dB SPL; CM Bs = 68 dB SPL; CAP Ex = 67 dB SPL). However, comparison of phase difference and amplitude ratios of 20JWA33 was similar to other animals with a minimal trauma rating; animals 20JWA28, 31 and 33 all showed a small CM difference (below −0.5π) and a large amplitude ratio. Animal 20JWA28 showed a small phase difference with a small amplitude ratio < 3.5 (3.2) and minimal severity of trauma. Animals 20JWA29 and 32 showed large phase differences <−0.5π and large amplitude ratios >3.5. Overall thresholds for these animals were similar (20JWA29: CM Ap = 43 dB SPL; CM Bs = 59 dB SPL; CAP Ex = 55 dB SPL and for 20JWA32: CM Ap = 47 dB SPL; CM Bs = 65 dB SPL; CAP Ex = 54 dB SPL. Moderate trauma was seen in 20JWA33, and minimal trauma 20JWA29 (Table 3). In animal 20JWA30 a large phase difference <−0.5π and small amplitude ratio <3.5 was observed; the difference in CM thresholds from El_Ap and El_Bs was small (−2 dB) and overall thresholds were low (CM Ap = 36 dB SPL; CM Bs = 38 dB SPL; CAP Ex = 43 dB SPL), indicating good cochlear health.

## 4 Discussion

We addressed CM phase differences and amplitude ratios from ECochG recordings to tone stimuli after cochleostomy and electrode array insertion using one extracochlear and two intracochlear electrodes in six normal-hearing guinea pigs. We found different severities of cochlear trauma as reflected by CAP and CM threshold shifts. In both CAP and CM the lowest thresholds and largest amplitudes were recorded with the most apically located intracochlear electrode. Differences in CM amplitudes and phases between the two intracochlear electrodes showed different combinations among the animals which may, as we argue, may provide additional information towards a conceptual framework to assess cochlear trauma after cochlear implantation (Harris et al., 2017; Weder et al., 2020; Sijgers et al., 2021; Bester et al., 2022; Scheperle et al., 2023).

### 4.1 Threshold and amplitude

The lowest CM and CAP thresholds and the largest CM and CAP amplitudes were most frequently recorded from intracochlear El_Ap. This first key finding could be explained by the fact that the recording electrode is positioned in closer proximity to potential residual generating hair cells towards the apex beyond the tip of the electrode, at least for the frequencies of 8 kHz and below (Bester et al., 2020; Scheperle et al., 2023; Sijgers et al., 2021; Calloway et al., 2014). Indeed, responses to 16 kHz were similar at the two intracochlear electrodes since these were located at the site most sensitive to 16 kHz (approximately 4 mm from round window; Tsuji and Liberman, 1997). However, varying patterns of CAP and CM thresholds between individual guinea pigs could be attributed to inter-animal differences in positioning of El_Ap and El_Bs in relation to hair cells and spiral ganglion neurons. In each guinea pig, the electrode array could have potentially twisted slightly differently during the insertion, causing variety in the radial position of the electrodes (Snyder et al., 2008). For both CM and CAP, our results showed similar significant main effects of frequency on thresholds and amplitudes. This could be explained by the fact that the amplitude of a CAP response mainly depends on the number of auditory nerve fibers firing synchronously, which is related to the number of functioning hair cells transmitting their response to the auditory nerve. The amplitudes of CAP and CM responses are therefore related to one another (Fitzpatrick et al., 2014; Forgues et al., 2014; Scott et al., 2016; Versnel et al., 1992). Lastly, a lower threshold and larger amplitude may be expected for CM and CAP to 16 kHz tones, because of the position of the recording electrodes near the tonotopic site of 16 kHz. However, thresholds were higher and amplitudes were smaller for the 16 kHz tones. This can be explained by trauma around the cochleostomy (Jwair et al., 2023) and by the normal sensitivity which is larger for 8 kHz than for 16 kHz in guinea pigs (Naert et al., 2019).

### 4.2 CM phase difference and amplitude ratio in relation to threshold and histological trauma

In a healthy cochlea, the CM phase difference between two electrodes follows the traveling wave difference (over 1 mm inter-electrode distance) and the amplitude ratio is approximately 1. As demonstrated by Bächinger et al. (2025), CM amplitudes are expected to be smaller in the case of basal trauma. As basal hair cell contributions are reduced, the responses of both electrodes will then reflect the same origin of more apically located hair cells, resulting in a larger amplitude ratio. When both electrodes share a common, more apically located CM source, spatial separation of functional hair cells is lost (Bester et al., 2020). Therefore the same biophysical mechanism also results in a smaller phase difference. The increase of amplitude ratio with a decrease of phase difference, as observed for five animals in the 2 kHz data (Figure 9A), could be explained by an increase of basal trauma. However, the 4 kHz data is more complex (Figure 9B). In our data, CM responses to 2 and 4 kHz showed longer phase difference and larger amplitudes at El_Ap than at the more basally located El_Bs.

The observed variations in CM amplitude and phase ratios can also be explained by changes in BM mechanics, in addition to changes in the quantity of viable hair cells. Bester et al. (2023) showed that BM fixation, either as a result of direct electrode–BM contact or secondary intracochlear fibrosis, is closely linked to mid-peak (MP) electrocochleographic patterns, which are characterized by basally shifted CM amplitude peaks and altered phase profiles. In the acute setting of our study, secondary intracochlear fibrosis did not yet play a role. BM mechanics could have been affected by electrode-BM contact. However, in our study the ratio between amplitudes from El_Bs/El_Ap was always larger than 1, indicating that El_Ap amplitude was larger than El_Bs amplitude in all cases (Figures 9A,B). With the short guinea pig electrode array positioned in the lower basal cochlear turn, a peak pattern similar to those found by Bester et al. (2023), i.e., a larger CM at the more basally located electrode, was not found.

**Figure 10:**
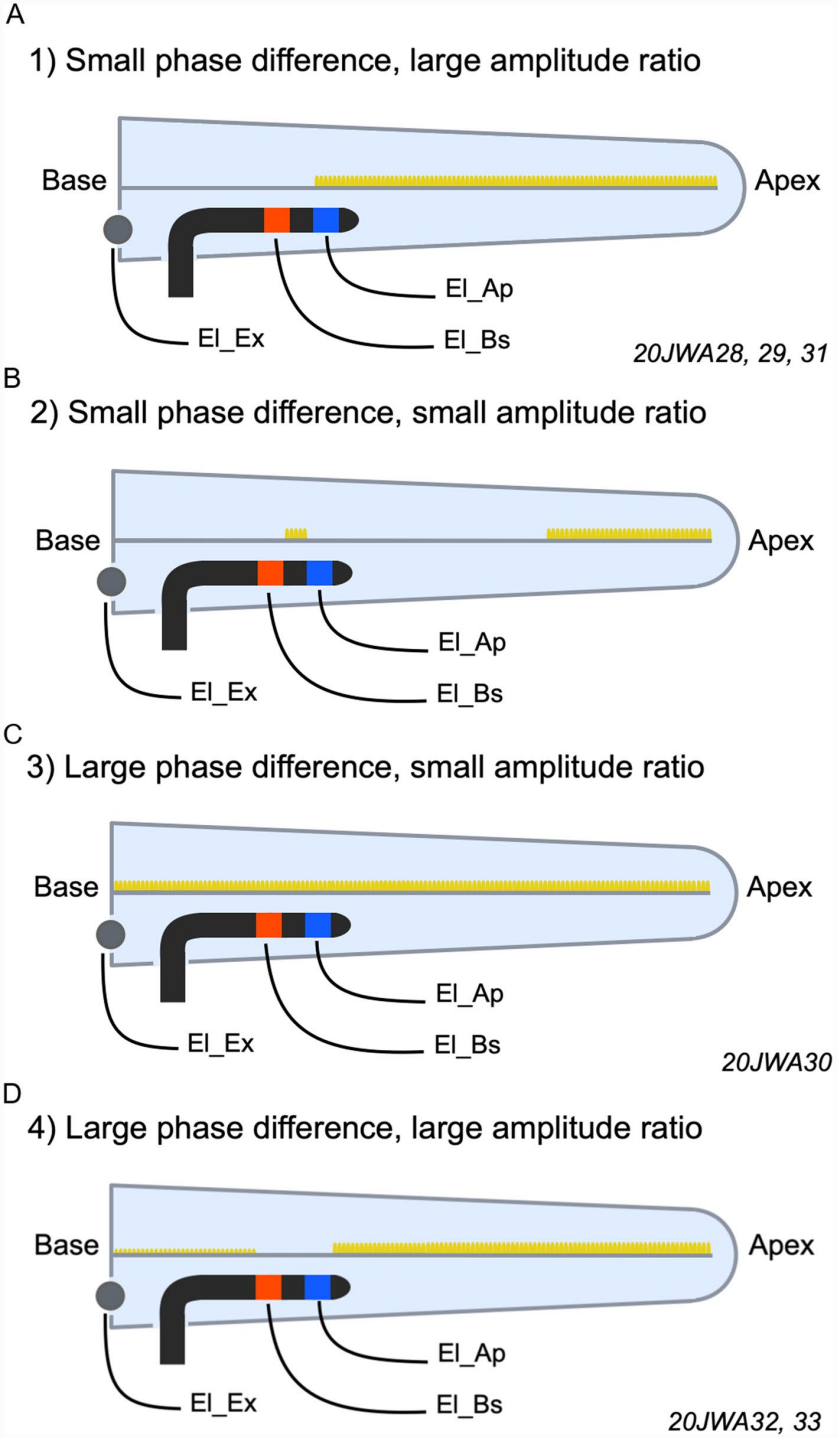
Schematic overviews of each combination of small or large CM phase difference and small or large CM amplitude ratio. Notably, these figures were not based on histological findings. **(A)** A small phase difference and large amplitude ratio (animals 20JWA28, 29, 31). **(B)** A small phase difference and a small amplitude ratio. **(C)** A large phase difference and a small amplitude ratio (animal 20JWA30). **(D)** A large phase difference and a large amplitude ratio (animals 20JWA32, 33). The yellow markers indicate areas along the basilar membrane (BM) containing functional hair cells. The empty areas along the BM (without yellow markers) indicate areas with dysfunctioning or lost hair cells. In **(D)** the basal end of the BM contains smaller yellow markers, indicating an area with less functional hair cells.

To assess local hair cell viability, we structured the discussion of our data around four different combinations of CM phase differences (small or large) with CM amplitude ratios (small or large) between both intracochlear electrodes. These combinations are all schematically depicted in Figures 10A–D. First, we describe each combination in detail, as each corresponds to a different spatial profile of cochlear trauma. Subsequently, we discuss some important considerations that are relevant to interpreting each combination of this conceptual framework.

The first combination, of a small CM phase difference and large amplitude ratio, would imply the responses at both electrodes originate from the same source, because hair cells near one of the recording electrodes are affected and are no longer significantly contributing to the CM (Bester et al., 2020). The much larger amplitude at El_Ap than at El_Bs indicates that the affected region is around El_Bs (Figure 10A). As a result, the responses recorded at electrodes El_Ap and El_Bs are both from the region adjacent to and more apical to El_Ap. Our data showed the combination of a small phase difference and large amplitude ratios in responses to 2 kHz for three animals (20JWA28, 29, 31; Figure 9A). According to the reasoning stated above, there should be damage to basal hair cells in these animals, even with minimal histological trauma (Table 3). As shown in a previous study, conducted on the same six guinea pigs, this trauma was inflicted by insertion of the electrode array rather than by cochleostomy, since CAP threshold shifts were minimal after cochleostomy (<5 dB), but substantial after insertion (>20 dB; Jwair et al., 2023). Also in response to 4 kHz, the same three animals showed a small phase difference (<0.5π) and a relatively large amplitude ratio. For each of the three animals the amplitude ratio for 4 kHz were smaller than for 2 kHz, possibly due to the fact that the responses to 4 kHz originate from an area located more basally than the area dominating the recordings to 2 kHz.

The second combination, of a small CM phase difference in combination with a small amplitude ratio, would suggest that hair cells near both of the recording electrodes are damaged. A cluster of functional hair cells located between El_Bs and El_Ap and/or hair cells apical beyond El_Ap, could be the common source for the CMs recorded at both electrodes, see Figure 10B. Viable hair cells between the two electrodes may give rise to responses at El_Ap and El_Bs, with very similar amplitudes and small ratios as a result. Other viable hair cells apical at some distance from El_Ap would yield a similar result. Our data did not contain clear examples of this category. 20JWA30 showed the combination of a small phase difference and a relatively small amplitude ratio at 2 kHz, but the data are close to the criteria cut off point (an amplitude ratio <3.5), which will be discussed in the next paragraph.

The third combination, being a large phase difference in combination with a small amplitude ratio, would imply a healthy condition of the cochlea near the recording electrodes. Each of the electrodes record responses from hair cells located near the electrode, and the traveling wave causes the difference between the recordings (Bester et al., 2020). Similar amplitudes indicate similar situations near both electrodes El_Ap and El_Bs: a similar number of surviving hair cells contributing to the CM responses, a similar distance between the electrodes and the viable hair cells, or a similar pattern of hair cell damage near both recording electrodes and between them (see Figure 10C). A good example for this combination is shown in the responses to 4 kHz in 20JWA30. This animal with minimal histological trauma showed low, similar thresholds from El_Ap and El_Bs (Bs: 38 dB SPL, Ap: 36 dB SPL). Although the responses of this animal to 2 kHz were of a different category (a small phase difference and small amplitude ratio), the responses were consistent with the 4 kHz data regarding substantial phase differences, small amplitude ratios and limited threshold differences (−12 dB).

The fourth and last combination, of a large phase difference and large amplitude ratio, would imply a condition similar to the large phase difference and small amplitude ratio category, i.e., functional hair cells at both electrode sites, but now with better survival near the apical site (causing larger CM amplitudes at the apical site than at the basal site). A large amplitude ratio can occur under different conditions. First, the hair cells close to El_Bs could be damaged with few functioning hair cells located more basally. Second, the electrode-hair cell distance can greatly differ, most likely with El_Ap closer to viable hair cells than El_Bs (depending on the site of the cochleostomy and the depth of insertion). An example of this combination is animal 20JWA32. CM thresholds at 4 kHz were 18 dB higher from El_Bs than from El_Ap, which reflects moderate histological trauma near El_Bs based on lateral wall damage at the cochleostomy site (see Table 3). Besides direct damage to hair cells, damage to the lateral could also damage the stria vascularis, resulting in decreased cochlear blood flow. This could in turn impair hair cell function in the basal turn. For this animal the amplitude ratios for 2 kHz were smaller than for 4 kHz, possibly caused by damage that affects the 4 kHz response more than the 2 kHz response.

Finally, phase differences longer than expected from the 1 mm inter-electrode distance can be seen as special cases within the fourth category. Animal 20JWA33 showed a phase difference of −*π* (corresponding to −0.25 ms) in the 2 kHz responses, which is twice as long as the expected difference of 0.11 ms (see “Methods”). This case with a longer than normal phase difference can be explained as follows. Hair cells around and between both electrodes are affected and therefore El_Bs records hair cells located more basally and El_Ap records the hair cells located more apically, which then explains the longer phase difference. The large amplitude ratio of 10 agrees with the threshold difference of 21 dB between El_Ap and El_Bs and indicates much larger trauma around the basal than around the apical site. The small phase difference seen in 20JWA33 implies hair cells near El_Bs responded poorly to 4 kHz, with the CM recorded at El_Bs reflecting mostly the same hair cells at El_Ap as a result.

### 4.3 Considerations

The data of the six animals followed three of the four categories of phase differences and amplitude ratios as described above. However, two of these categories suggesting basal hair cell trauma (1 and 4), could not be confirmed with our histological findings. Kang et al. (2010) and Tanaka et al. (2014) found similar results, as they also reported CAP threshold shifts without quantifying damage to IHCs and OHCs in histological sections. These findings could be explained by histological assessment being based on midmodiolar sections, only showing damage in the middle of the basal turn. This emphasizes the importance of complete cochlear sampling, with for example light sheet fluorescence microscopy, which allows investigation of the anatomical relationships between intracochlear components such as hair cells and the BM in the whole cochlea (Hutson et al., 2021). Another explanation could be that implantation of the electrode array may cause displacement and consequently a change in BM mobility and mechanics, which results in disrupted functioning of hair cells (DeMason et al., 2012). BM mechanical alterations, in an acute setting most likely due to electrode-BM contact, can reduce the local traveling-wave delay, reducing phase differences. Local focusing of the vibrations of the BM adjacent to the fixation site, can modify amplitude ratios (Bester et al., 2023). Therefore, we highlight that our aim was to introduce an conceptual framework for assessing trauma based on phase differences and amplitude ratios, rather than to assert that this framework represents the optimal approach of analysis of CM responses to assess damage. Threshold shifts could also be explained by a drop in endocochlear potential (Tanaka et al., 2014). The endocochlear potential may drop due to several reasons. First, piercing of the basal BM by the array, whether or not visible in the midmodiolar section, may cause mixture of endolymph and perilymph, resulting in a disrupted hair cell membrane potential (Choudhury et al., 2011). Furthermore, insertion damage to the stria vascularis, resulting in deterioration of cochlear blood circulation, could also cause the endocochlear potential to drop, as Tanaka et al. (2014) reported a correlation between impaired blood circulation in the cochlea and threshold shifts after implantation.

## 5 Conclusion

Differences in ECochG responses from two intracochlear electrodes can be applied as relative measures to assess the degree of cochlear damage caused by cochlear implantation surgery. In addition to conventional thresholds, CM phase differences and amplitude ratios may provide spatial information of hair cell trauma.

## Data Availability

The original contributions presented in the study are included in the article/Supplementary material, further inquiries can be directed to the corresponding author.
